# *In silico* tools for accurate HLA and KIR inference from clinical sequencing data empower immunogenetics on individual-patient and population scales

**DOI:** 10.1093/bib/bbaa223

**Published:** 2020-09-17

**Authors:** Jieming Chen, Shravan Madireddi, Deepti Nagarkar, Maciej Migdal, Jason Vander Heiden, Diana Chang, Kiran Mukhyala, Suresh Selvaraj, Edward E Kadel, Matthew J Brauer, Sanjeev Mariathasan, Julie Hunkapiller, Suchit Jhunjhunwala, Matthew L Albert, Christian Hammer

**Affiliations:** Department of Bioinformatics and Computational Biology; Department of Cancer Immunology; Department of Cancer Immunology; Roche’s Global IT Solution Centre; Department of Bioinformatics and Computational Biology; Department of Human Genetics; Department of Bioinformatics and Computational Biology; Roche/Genentech’s Biosample & Repository Management; Department of Oncology Biomarker Development; Data Science at Maze Therapeutics; Department of Oncology Biomarker Development; Department of Human Genetics; Department of Bioinformatics and Computational Biology; Immunology & Infectious Diseases; Departments of Cancer Immunology and Human Genetics

**Keywords:** HLA, KIR, immunogenetics, whole-genome sequencing, imputation, clinical sequencing

## Abstract

Immunogenetic variation in humans is important in research, clinical diagnosis and increasingly a target for therapeutic intervention. Two highly polymorphic loci play critical roles, namely the human leukocyte antigen (HLA) system, which is the human version of the major histocompatibility complex (MHC), and the Killer-cell immunoglobulin-like receptors (KIR) that are relevant for responses of natural killer (NK) and some subsets of T cells. Their accurate classification has typically required the use of dedicated biological specimens and a combination of *in vitro* and *in silico* efforts. Increased availability of next generation sequencing data has led to the development of ancillary computational solutions. Here, we report an evaluation of recently published algorithms to computationally infer complex immunogenetic variation in the form of HLA alleles and KIR haplotypes from whole-genome or whole-exome sequencing data. For both HLA allele and KIR gene typing, we identified tools that yielded >97% overall accuracy for four-digit HLA types, and >99% overall accuracy for KIR gene presence, suggesting the readiness of *in silico* solutions for use in clinical and high-throughput research settings.

## Introduction

The classical human leukocyte antigen (HLA) gene complex on chromosome 6, and the killer-cell immunoglobulin-like receptor (KIR) genes on the leukocyte receptor complex on chromosome 19 are complex genomic loci that have been known to be difficult to genotype accurately. With the rapidly emerging treatment approaches in the fields of cancer immunotherapy [1, 2] and autoimmunity [3], the accurate characterization of a patient’s immunogenetic composition in the HLA and KIR regions is becoming more clinically important.

Classical HLA proteins play an important role in presenting peptides derived from self, tumor or microbial antigens. They display an extreme amount of allelic polymorphism, as a result of pathogen-driven and balancing selection [4]. Much research has shown these HLA variants to be strongly associated with multiple immune and nonimmune phenotypes in the fields of cancer [5, 6], autoimmunity [3, 7], neurodegeneration [8] and infectious diseases [3, 7]. In the clinic, achieving matched classical HLA alleles between the donor and recipient is critical for organ and stem cell transplantation, such that HLA typing and matching have been integrated as part of standard clinical protocols for decades [9–11]. HLA typing has also become increasingly important in diagnostics and clinical practice. For example, several approved drugs carry labels indicating increased risk for adverse events for carriers of specific HLA alleles [12–14].

KIR proteins are receptors for classical HLA class I ligands, and are predominantly expressed on natural killer (NK) cells. In contrast to most HLA genes, the genes coding for KIR display extensive copy number polymorphism [15], in addition to considerable allelic variation [16] for each gene. KIR have shown significant associations with disease phenotypes, mainly in the fields of infectious diseases, autoimmunity, inflammatory diseases and cancer [17]. Associations were found for both single KIR genes, and when considering their interactions with specific HLA molecules. HLA–KIR interactions were demonstrated to predict the risk of organ rejection after kidney transplantation, suggesting a clinical use case for KIR typing [18, 19]. KIR proteins are also known to be important codeterminants of NK cell education, which is in part mediated through their interactions with different HLA molecules. Such interactions significantly define the heterogeneity of NK cell responsiveness and their sensitivity to inhibition by HLA across individuals [20, 21]. As such, KIR proteins play a critical role in the recognition of ‘missing-self’ phenotypes in infected or tumor cells, which are typically defined by the loss or down-regulation of HLA class I cell surface expression [22].

For research purposes, genotyping arrays covering single nucleotide polymorphisms (SNPs) across the genome have been used to impute HLA and KIR types. However, they require statistical imputation methods to disentangle the complex linkage disequilibrium (LD) between SNPs and HLA or KIR types [23, 24]. These methods also rely on the availability of ancestry-specific or multiancestry reference panels that can be difficult to obtain, especially for populations not well represented in genomic data sets [25]. In clinical diagnostics, dedicated immunogenetics laboratory solutions to HLA and KIR genotyping are being continually developed [26]. Initial molecular typing technologies were low throughput and/or probe-based assays [27, 28]. In recent years, high-throughput next generation sequencing (NGS) has become increasingly affordable [29]. This has enabled the prevalent use of short-read NGS, namely whole-genome sequencing (WGS) and whole-exome sequencing (WES), in the clinic [30, 31]. Although well-validated bioinformatics pipelines have been implemented to detect millions of genetic variants from the available clinical sequences [32, 33], they are typically employed uniformly to the entire genome or exome, and can be ineffective at particular genomic loci that are highly polymorphic, such as the HLA and KIR regions. Dedicated *in silico* typing tools that use NGS data and specifically target the HLA or KIR genes could be a cost-effective and efficient alternative to traditional laboratory HLA or KIR typing methods. Although such NGS-based approaches do not require linkage-disequilibrium-based statistical imputation for genotyping (because the sequencing reads directly contain the information to define e.g. the HLA allele status), they do require the use of comprehensive databases that capture the diversity and complexity of these genomic loci for alignment (as opposed to single reference genomes).

Despite their biological significance and many practical advantages, the development of clinically ready *in silico* HLA and KIR typing have thus far been largely hampered by the genetically complex and highly polymorphic nature of the two regions [7, 34]. Bauer *et al.* provided an extensive evaluation of available HLA genotyping tools in 2016 [35]. They showed that the concordance of HLA typing with their gold standard dataset is generally low, with the best accuracy for a combined HLA I and II genes at 73%. Since then, a new generation of *in silico* HLA genotyping tools has been created, which showed much promise for high accuracy. Here, we conducted a survey of the recent HLA and KIR typing capabilities for potential scaling and readiness in clinical applications. We evaluated available computational HLA inference tools by comparing the inferred HLA alleles from WES and WGS data to a gold standard dataset, which was generated using a commercial dedicated typing method. We also assessed and validated a recently published KIR method by Roe and Kuang [36], which can be used to infer KIR gene presence and absence from WGSdata.

## Methods

### Generation of a gold standard HLA reference dataset

The gold standard reference dataset contains 56 patient samples. Genomic DNA was extracted from 1 ml of EDTA whole blood on the Roche MagNA Pure 96 system using the MagNA Pure 96 DNA and Viral NA Large Volume Kit. They were then sent to LabCorp (Burlington, NC, USA) for sequence-based, two-field HLA genotyping, using accepted scientific standards meeting the accreditation requirements of the American Society for Histocompatibility and Immunogenetics (ASHI) and the College of American Pathologists (CAP). HLA class I genotypes of *HLA-A, -B* and *-C* were determined using a combination of long-range sequencing from PacBio (Menlo Park, CA, USA) RSII and Sanger sequencing. Class II genotypes for *HLA-DPA1, -DPB1, −DQA1, -DQB1, -DRB1, -DRB3, -DRB4, -DRB5* were determined using a combination of long-range sequencing from PacBio RSII, and NGS from Illumina (San Diego, CA, USA) TruSight and MiSeq technologies.

### Generation of a KIR reference dataset

Genotyping of KIR alleles was performed for 72 patients using the LinkSeq KIR kit according to manufacturer’s user guide (Catalog No: 5358R, One Lambda, Canoga Park, CA, USA). Briefly, the human genomic DNA was amplified and melt curves were collected on a real-time PCR instrument (QuantStudio 5 system, Thermo Fisher Scientific, Waltham, MA, USA). The data were exported to SureTyper software 6.1.2 (One Lambda) for interpretation and reporting of the genotype.

### Whole-genome and whole-exome sequencing

WGS and WES were performed for the samples in the HLA and KIR reference datasets, on Illumina HiSeq instruments using paired-end reads of 150 bp. The bait set used for WES was the Illumina Nextera Exome Kit with 38 megabases target territory (29 megabases baited). The WG samples were sequenced at an average of 37× coverage (range = 31–50×). For WES, all samples were sequenced at an average of 80× coverage (range = 70-99×), and passed QC criteria of 85% of targeted bases at 20× or greater coverage (±5%). FASTQ files were generated for each WG and exome, and were used as direct inputs for each tool, where appropriate. For tools that required BAM files as inputs, the FASTQ files were processed according to a workflow built using the Genome Analysis Toolkit (GATK) best practices from 2015 and GATK v3.5 [33]. Briefly, it includes read alignment using bwa 0.7.15 (bwa mem) with GRCh38 as the reference genome, duplicate marking using Picard tools v2.9, indel realignment using GATK v3.5, and then base quality score recalibration using GATK 3. For bwa, except for the ‘-M’ flag, all the default options were used. Individual-level genetic data for this study cannot be made publicly available due to consent restrictions.

### Selection and configuration of HLA typing tools

For WGS and WES HLA genotyping, we specifically selected tools that (i) are recently published, (ii) are easy to install and implement, (iii) have the ability to work with WGS and WES data (iv) and could genotype both HLA I and II genes for potential clinical use. These included HLA*PRG:LA [37], xHLA v1.2 [https://github.com/humanlongevity/HLA] [38], HLA-HD v1.2 [https://www.genome.med.kyoto-u.ac.jp/HLA-HD/] [39], and HISAT2 v2.1 [https://ccb.jhu.edu/software/hisat2/index.shtml] [40]. As a comparison to a more widely used tool, we also assessed results from Polysolver v1.0 [41]. Polysolver has been widely utilized in many benchmarking and research studies, and has been shown to be one of the more accurate genotyping tools, albeit only for classical HLA class I genes [42–44]. We summarized each tool’s main algorithm of genotyping HLA alleles, unique characteristics, various features and implementation idiosyncrasies in Table 1. For more in-depth discussion on their algorithms, please refer to their respective publications and corresponding supplementary materials; for more details in implementation, please refer to related documentation (listed in Table 1).

**Table 1:** Overview of *in silico* HLA genotyping tools used in the study

	Polysolver	HLA*PRG:LA	xHLA	HLA-HD	HISAT2
Data type:					
Designated data modality	WES, WGS	WES, WGS	WES, WGS	WES, WGS, RNA-seq	WES, WGS, RNA-seq
Type of sequencing data reported	Short-read	Short-read, long-read	Short-read	Short-read	Short-read
Dependencies:					
Aligner	Novoalign	BWA MEM	Diamond	Bowtie2	HISAT2
Other tools (beyond Linux apps)	Java, Samtools, Picard	Boost, Bamtools, Samtools, Picard	Samtools, BWA, Bedtools	–	Samtools
Databases:					
IMGT database version bundled with software	V3.10.0	Unknown	Unknown	V3.15.0	V3.26.0
Can IMGT database used be updated?	Unknown	Unknown	Unknown	Yes	Unknown
Allows use of population allele frequencies?	Yes (optional)	No	No	Yes (conditional)	No
Input:					
Preprocessing required before actual implementation?	No	No^*^	Yes	No	Yes^*^
Input file format	BAM	BAM	BAM	FASTQ	FASTQ
Algorithm:					
Alignment to IMGT database reference	DNA alignment	DNA alignment, graph-based reference	Protein alignment, perfect match to core exons, then iterative refinement using noncore exons	DNA alignment, perfect match to core exons, then to noncore exons, and up to two mismatches in introns	DNA alignment, graph-based reference
Statistical algorithm to determine calls	Bayesian classification approach per allele	Maximum likelihood per allele	Integer linear programming per solution set of alleles	Maximum score per top pair of alleles of a gene	Expectation—maximization algorithm to find maximum likelihood estimate per allele
Output:					
Results/output file format	SSV	TSV	JSON	SSV	Extracted from log file
Classical HLA genes beyond HLA I (*HLA-A, -B, -C*)	–	*HLA-DQA1/B1, -DRB1*	*HLA-DPB1, -DQB1, -DRB1*	*HLA-DPA1/B1, −DQA1/B1, −DRA/B1/B3/B4/B5*	*HLA-DQA1/B1, -DRB1*
Maximum resolution	Four-field	G group	Two-field	Three-field	Four-field
Ability to detect novel alleles	No	No	No	No	No
Other characteristics:					
Average time taken for one sample in this study (preprocessing+HLA typing)	WES: 1.5 h WGS: 4 h	WES: 1.5 h WGS: 2 h	WES: 7 + 7 min WGS: 2 h +10 min	WES: 1 h WGS: 25 h	WES: 3 h +7 min WGS: 33 h +16 min
License	BSD-style license, but Novoalign is only free for educational, nonprofit use	GNU GPL v3.0	Free for research use	Free for research use	GNU GPL v3.0
Troubleshooting	Author correspondence	Github	Github	Author correspondence	Github
Best available documentation^a^	Website, author correspondence	Readme, blog post	Readme	Website, PMID: 29858813	Readme, website, paper supplementary
PMID	26,372,948	27,792,722	28,674,023	28,419,628	31,375,807
Source	Broad Institute	Wellcome Trust	Human Longevity, Inc.	University of Kyoto	John Hopkins University

A user-defined graph derived from a reference genome can be optionally constructed prior to actual HLA genotyping for both HLA*PRG:LA and HISAT2. Although this can add a considerable amount of time (several hours), it only needs to be done once for an entire cohort. In this study, we used the references that came with the tools (please refer to Supplementary Table 1). For HISAT2, even though we used the graph provided, there is still an additional preprocessing step prior to actual HLA genotyping.

^a^Polysolver: https://software.broadinstitute.org/cancer/cga/polysolver_run

HLA*PRG:LA: Github - https://github.com/DiltheyLab/HLA-LA; Blog - https://genomeinformatics.github.io/HLA-PRG-LA/

xHLA: https://github.com/humanlongevity/HLA

HLA-HD: https://www.genome.med.kyoto-u.ac.jp/HLA-HD/

HISAT2: Github - https://github.com/DaehwanKimLab/hisat2; Website - http://ccb.jhu.edu/hisat-genotype/index.php/Main_Page

There are some tools that require a BAM file format, i.e. read alignment to a reference genome. For xHLA and Polysolver, FASTQs were aligned using BWA MEM to hg38 to produce BAMs. They then require further preprocessing. For xHLA, BAMs were preprocessed with an additional bash script provided by the authors [https://github.com/humanlongevity/HLA/blob/master/bin/get-reads-alt-unmap.sh]. Polysolver, by default, hardcoded hg18 or hg19 genomic coordinates for read extraction from chromosome 6 in the BAM files prior to HLA genotyping. In order to overcome this limitation, we modified the code to include the use of BAMs that are aligned to hg38 as well. No other modifications were made to the code. For HLA-HD and HISAT2, the raw FASTQs were used directly. All the tools were run using default settings, and with the HLA/IMGT databases versions that came with the respective tools. For the exact run parameters, please refer to Supplementary Table 1.

### Evaluation of HLA typing tools

The tools typically give two alleles per HLA gene, but we do see in occasions, albeit rare, in our study where the algorithm provided more than one pair of possible alleles, and often in descending order of significance. For any results that offer more than two alleles, we only took the top two inferred HLA alleles; we assumed a diploid germline genome.

Each *in silico* HLA typing tool was evaluated by two accuracy metrics. (i) The ‘allele concordance’ with the gold standard dataset, which counts the number of concordant HLA alleles called by the tool when compared with the reference HLA genotypes obtained from LabCorp, for the classical HLA I and II genes. Its accuracy was defined as the quotient of the number of concordant calls and the sum of the number of concordant plus discordant calls. (ii) The ‘full-sample concordance’ counts the number of samples that have perfect concordance with the gold standard dataset. Its accuracy was defined as the quotient of the number of samples with full concordance and the total number of samples that were successful in the run for each tool. For uniformity, results from the tools were converted to four-digit resolution before evaluation, except for HLA*PRG:LA, which can only perform genotyping at G group resolution. A G group consists of HLA alleles that have identical nucleotide sequences in the exons coding for the peptide binding domain (exons 2 and 3 for HLA class I and exon 2 for class II), whereas a P group contains all HLA alleles that have the same amino acid sequence for the exons coding for the peptide binding domain. A minimum of P group resolution or higher (including G group, four-digit/two-field resolutions), is usually considered ‘high resolution typing’ and therefore clinically relevant [45]. For HLA*PRG:LA, gold standard results were first converted to G groups before evaluation. G group information was obtained from the IMGT/HLA database [http://hla.alleles.org/alleles/g_groups.html].

We split the evaluation of the tools’ accuracy into three categories: (i) HLA I, consisting of classical HLA I genes *HLA-A*, *-B* and *-C*, (ii) HLA IIa, consisting of classical HLA II genes *HLA-DPA1*, *-DPB1*, *−DQA1*, *-DQB1* and *-DRB1* and (iii) HLA IIb, consisting of a second group of classical HLA II genes, *HLA-DRB3*, *-DRB4* and *-DRB5* (Table 2). We created a second category (HLA IIb) for additional DR genes because only HLA-HD currently genotypes them. Additionally, since every individual carries a variable copy number of the three genes *DRB3, DRB4* or *DRB5* that is highly dependent on the *DRB1* genotype [46], a no-call by the computational tool is considered concordant with the gold standard results if it is not also identified. Some tools cannot genotype the full set of the classical class II genes, thus we also provided the accuracy with respect to each class II gene (Table 3).

**Table 2:** Overall evaluation results for HLA typing using WES and WGS. HLA genes are categorized into classical class I genes (*A, B* and *C*), IIa genes (*DPA1, DPB1, DQA1, DQB1* and *DRB1*), and IIb genes (*DRB3, DRB4* and *DRB5*). The class IIa genes that each tool can genotype differ: HLA-HD can infer all of the above; xHLA can only infer *DPB1, DQB1* and *DRB1*; HISAT2 and HLA*PRG:LA only *DQA1, DQB1* and *DRB1*. Note that HLA*PRG:LA was evaluated based on the G group resolution. Results from the older and well-utilized Polysolver were provided as an additional source of comparison for HLA I genes. For each category, we also provided two accuracy metrics, (i) allele concordance, computing the total number of alleles that are concordant with the gold standard data, and (ii) full-sample concordance, computing the number of samples that have perfect concordance with the gold standarddata

Method	Allele or full-sample concordance	WES concordance (%)			WGS concordance (%)		
		I^b^	IIa	IIb	I^b^	IIa	IIb
Polysolver	Allele	328/336(97.6)	–	–	330/336(98.2)	–	–
	Sample	49/56(87.5)	–	–	51/56(91.1)	–	–
HLA*PRG:LA (G groups only)	Allele	333/336(99.1)	335/336(99.7)	–	333/336(99.1)	335/336(99.7)	–
	Sample	53/56(94.6)	55/56(98.2)	–	53/56(94.6)	55/56(98.2)	–
xHLA	Allele	156/330(47.2)^a^	187/330(56.7)^a^	–	327/336(97.3)	327/336(97.3)	–
	Sample	6/55(10.9)	7/55(12.7)	–	49/56(87.5)	50/56(89.3)	–
HLA-HD	Allele	334/336(99.4)	552/560(98.6)	329/336(97.9)	334/336(99.4)	557/560(99.5)	328/336(97.6)
	Sample	54/56(96.4)	48/56(85.7)	50/56(89.3)	54/5696.4)	53/56(94.6)	49/56(87.5)
HISAT2	Allele	332/336(98.8)	310/336(92.3)	–	334/336(99.4)	324/336(96.4)	–
	Sample	52/56(92.9)	42/56(75)	–	54/56(96.4)	49/56(87.5)	–

^a^1 sample failed to run in xHLAWES.

^b^Included miscalls for a novel *HLA-C* allele at four-digit resolution. All tools matched the LabCorp result at two-digit resolution correctly.

**Table 3:** Evaluation results by HLA II genes for HLA typing using WES and WGS. Note that HLA*PRG:LA was evaluated based on the G group resolution, not at four-digit resolution. For each gene, we provided two accuracy metrics, (i) allele concordance, computing the total number of alleles that are concordant with the gold standard data, and (ii) full-sample concordance, computing the number of samples that have perfect concordance with the gold standarddata

HLA II gene	Allele or full-sample concordance	Polysolver (%)		HLA^*^PRG:LA (%)		xHLA (%)		HLA-HD (%)		HISAT2 (%)	
		WES	WGS	WES	WGS	WES^a^	WGS	WES	WGS	WES	WGS
*A*	Allele	112/112(100)	112/112(100)	110/11298.2)	110/112(98.2)	60/110(54.5)	111/112(99.1)	112/112(100)	112/112(100)	112/112(100)	112/112(100)
	Sample	56/56(100)	56/56(100)	54/56(96.4)	54/56(96.4)	19/55(34.5)	55/56(98.2)	56/56(100)	56/56(100)	56/56(100)	56/56(100)
*B*	Allele	107/112(95.5)	109/112(97.3)	112/112(100)	112/112(100)	46/110(41.8)	107/112(95.5)	111/112(99.1)	111/112(99.1)	110/112(98.2)	111/112(99.1)
	Sample	51/56(91.1)	53/56(94.6)	56/56(100)	56/56(100)	11/55(20)	50/56(89.3)	55/56(98.2)	55/56(98.2)	54/56(96.4)	55/56(98.2)
*C* ^b^	Allele	109/112(97.3)	109/11297.3)	111/112(99.1)	111/112(99.1)	50/110(45.5)	109/112(97.3)	111/112(99.1)	111/112(99.1)	110/112(98.2)	111/112(99.1)
	Sample	53/56(94.6)	53/56(94.6)	55/56(98.2)	55/56(98.2)	10/55(18.2)	54/56(96.4)	55/56(98.2)	55/56(98.2)	54/56(96.4)	55/56(98.2)
*DPA1*	Allele	–	–	–	–	–	–	112/112(100)	112/112(100)	–	–
	Sample	–	–	–	–	–	–	56/56(100)	56/56(100)	–	–
*DPB1*	Allele	–	–	–	–	70/110(63.6)	109/112(97.3)	110/112(98.2)	110/112(98.2)	–	–
	Sample	–	–	–	–	20/55(36.3)	54/56(96.4)	54/56(96.4)	54/56(96.4)	–	–
*DQA1*	Allele	–	–	112/112(100)	112/112(100)	–	–	106/112(94.6)	111/112(99.1)	111/112(99.1)	110/112(98.2)
	Sample	–	–	56/56(100)	56/56(100)	–	–	50/56(89.3)	55/56(98.2)	55/56(98.2)	55/56(98.2)
*DQB1*	Allele	–	–	111/112(99.1)	112/112(100)	53/110(48.2)	107/112(95.5)	112/112(100)	112/112(100)	111/112(99.1)	110/112(98.2)
	Sample	–	–	55/56(98.2)	56/56(100)	16/55(29.1)	50/56(89.3)	56/56(100)	56/56(100)	55/56(98.2)	55/56(98.2)
*DRB1*	Allele	–	–	112/112(100)	111/112(99.1)	64/110(58.2)	111/112(99.1)	112/112(100)	112/112(100)	88/112(78.6)	104/112(92.9)
	Sample	–	–	56/56(100)	55/56(98.2)	20/55(36.3)	55/56(98.2)	56/56(100)	56/56(100)	43/56(76.8)	50/56(89.3)
*DRB3*	Allele	–	–	–	–	–	–	110/112(98.2)	111/112(99.1)	–	–
	Sample	–	–	–	–	–	–	54/56(96.4)	55/56(98.2)	–	–
*DRB4*	Allele	–	–	–	–	–	–	110/112(98.2)	108/112(96.4)	–	–
	Sample	–	–	–	–	–	–	55/56(98.2)	53/56(94.6)	–	–
*DRB5*	Allele	–	–	–	–	–	–	109/112(97.3)	109/112(97.3)	–	–
	Sample	–	–	–	–	–	–	53/56(94.6)	53/56(94.6)	–	–

^a^1 sample failed to run in xHLAWES.

^b^Included miscalls for a novel *HLA-C* allele at four-digit resolution. All tools matched the LabCorp result at two-digit resolution correctly.

### Evaluation of KIR typing with kpi and interpretation of results

For inference of KIR gene presence or absence, we evaluated kpi [https://github.com/droeatumn/kpi, downloaded 11 March 2020]. An earlier version of the software was recently presented in a preprint and did not provide a validation of its accuracy when compared to qPCR-based dedicated KIR typing methods [36]. Kpi requires WGS FASTQ files as input data and outputs a presence/absence call for each KIR gene, as well as possible combinations of KIR haplotypes according to a provided list of reference haplotypes [https://github.com/droeatumn/kpi/blob/master/input/haps.txt]. Each KIR gene can in principle be characterized by copy number and allelic variation. A KIR haplotype determines the order and presence of single KIR genes [47, 48]. However, kpi only detects presence or absence of each KIR gene, not allele status or copy number. As a result, the calls for haplotype pairs can be ambiguous (due to differences in copy number of present genes), but the presence of single KIR genes can be resolved [36]. As such, KIR typing with kpi, albeit coarse, is still useful because many associations have been reported on haplotype or gene level. Furthermore, interactions of KIRs with their HLA ligands are usually defined at the KIR gene level [17, 22]. It should be noted though, that the KIR genes do show extensive allelic polymorphism that can still have an effect on such defined interactions [34, 49].

### Inference of HLA–KIR interactions

NK cell inhibiting as well as activating KIR interactions with their HLA class I ligands were defined according to Pende *et al*. [18] Briefly, some KIR interact with groups of four-digit HLA alleles according to specific HLA amino acid residues. *HLA-B* alleles were classified as either *Bw4* or *Bw6* according to amino acid positions 77–83. *HLA-C* alleles were assigned C1 or C2 status based on amino acid position 80 [18]. Other interactions were defined between KIR and specific two-digit or four-digit HLA alleles (e.g. A*03 – KIR3DL2).

## Results

### High accuracy for HLA I and II typing with current gold standard WES and WGS data

We selected HLA genotyping tools to infer HLA identities of 56 patients from the EXCELS (NCT00252135) [50] and AVANT (NCT00112918) [51] clinical trials. The inferred HLA types were then compared to results from the gold standard reference dataset. The diversity of HLA alleles is imperative in the evaluation of HLA genotyping tools, as it allows testing of the tools against different alleles. Despite the limited size of our dataset, the diversity of HLA class I alleles for each HLA gene in our samples is highly comparable to the publicly available, ethnically varied and sequencing-derived 1000 genomes/HapMap validation set generated by Ehrlich *et al.* [52] (Supplementary Table 1).

From the results consolidated in Table 2, all the selected genotyping tools perform generally well, at an allelic accuracy of >90% for most of the class I and II gene categories, except for xHLA on WES class I and IIa genes. xHLA demonstrated uncharacteristically low accuracy for our WES data for both classes I and II, when compared to both reported performance [38] and the other tools. HISAT2 and HLA-HD performed comparably for the HLA I genes *A*, *B* and *C* for both WES and WGS data, at >98.8% accuracy. Overall, for class II genes, HLA-HD is consistently the most accurate HLA typing tool for both WES and WGS data, at >97.6% accuracy for *DRB3*, *DRB4* and *DRB5*, and >98.6% accuracy for the *DPA1/B1*, *DQA1/B1* and *DRB1* genes. Moreover, it also provides the widest range of HLA II genes, with the ability to genotype all the classical class II genes (including *HLA-DRA, -DRB3, -DRB4* and *-DRB5*), whereas other tools are restricted to a subset of classical class II alleles. However, HLA-HD has lower accuracy for *DQA1* when working off WES data, compared to HISAT2 (Table 3).

Although we observed similar or increased accuracy when comparing results from WGS to WES data from the same tool (Table 2), WGS and WES miscalls were not always the same. This is evident when assessing accuracy at the gene level. For example, the increase in overall accuracy of HISAT2 when using WGS data for identifying class II alleles was mainly due to a lower *HLA-DRB1* accuracy when using WESdata.

We next focused on the top performant methods, HLA-HD and HISAT2. Notably, the number of miscalls in HLA I genes was too low to characterize patterns or biases (Table 2; only a maximum of four miscalls). For class II genes, HISAT2 showed the highest number of *HLA-DRB1* miscalls when using WES data (Table 3). This is largely due to a higher number of missing calls generated in *HLA-DRB1* by HISAT2 in WES than WGS data. For WES data, 11/13 samples have no-calls in both *HLA-DRB1* alleles, and for WGS, 2/6 samples. No-calls are quite rare in the results of the other tools (except xHLA in WES data). Coupled with the fact that similar observations occur in both WGS and WES (albeit to different degrees), the no-calls might indicate a larger issue with HISAT2 in calling *HLA-DRB1* alleles. We found that there is an enriched number of no-calls originating from carriers of DRB1*15:01 compared to samples with non-DRB1*15:01 alleles in both WES (Fisher’s test *P* = 0.008) and WGS (*P* = 0.04). By contrast, HLA-HD miscalled mostly in the class IIb genes, where it failed to discriminate the highly similar alleles of the paralogous genes *HLA-DRB3*, *-DRB4* and *-DRB5*. There was no obvious bias in miscalls of homozygous genes in the HLA-HD and HISAT2 results (Supplementary Table 2). We note that most of HLA-HD miscalls still called an allele in the same G group as the anticipated allele. In particular, almost all the miscalls in *DQA1* (5/6 of DQA1 miscalls and 5/8 of total miscalls) were made when they were called as DQA1*03:01 and the anticipated calls were DQA1*03:02 and DQA1*03:03; all three alleles are in the same G group. For HISAT2, many of the miscalls in class II genes were due to missing calls, i.e. calls that the tool was not able to assign an allele atall.

**Figure 1:**
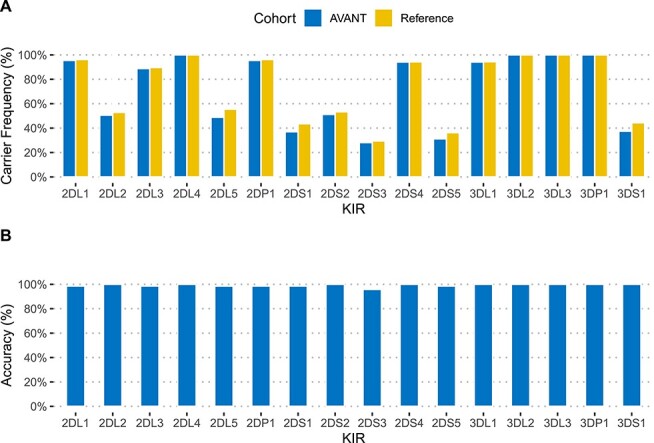
KIR gene carrier frequencies and accuracy of kpi typing. (**A**) KIR gene presence for AVANT patients (*N* = 824) was inferred from kpi haplotype predictions, and compared to published frequencies for an English reference cohort (*N* = 584). (**B**) For 72 AVANT patients typed with kpi, KIR typing with a qPCR-based method (LinkSeq) was performed to assess typing accuracy.

Additionally, we used a metric, we called ‘full-sample concordance’, where we computed the proportion of samples that were in full concordance with our reference dataset, for the three HLA gene categories and also for each HLA gene. We observe that most of the full-sample concordance exceeded 80% in both WES and WGS, which is expected given the high allelic concordance. This observation also implies that there is little sample bias in general, i.e. errors do not originate from the NGS data of specific samples. An exception is the WES results of HISAT2 in inferring HLA alleles that are in class IIa at 75%. This is primarily due to the enriched number of no-calls in *HLA-DRB1* genes by HISAT2 in WES data, as described above. Since DRB1*15:01 is common in European samples, the high number of no-calls resulted in a decreased full-sample concordance despite high allelic concordance.

### High accuracy for identifying KIR gene absence/presence

We used kpi to infer KIR gene presence for 824 patients with available WGS data from the AVANT trial (NCT00112918) [51]. We found that the gene carrier frequencies were very similar to those of published KIR gene carrier frequencies from an English cohort of Caucasoid ethnicity [53]. (Figure 1A). The software was unable to predict possible haplotype pairs for 3% (*n* = 25) of cases.

We selected 72 of these 824 patients to perform qPCR-based KIR typing, based on DNA sample availability and the diversity of kpi-predicted KIR haplotypes. These patients are different from those selected for the HLA gold standard data because of limited availability of DNA. For this selection, we also included 8 of the 25 patients that had yielded uninterpretable haplotype combinations usingkpi.

Almost 99.2% of kpi gene presence/absence calls were correct when compared to our qPCR-based reference (range of 95.8–100% for the 16 tested genes, Figure 1B, Supplementary Table 3). When excluding the four framework genes *KIR3DL3*, *KIR3DP1*, *KIR2DL4* and *KIR3DL2*, which were invariable in our dataset and are present in most common haplotypes, the accuracy for the 12 remaining genes was 99.0%. Three patients were typed with one error each (KIR2DS3), and one patient with six errors. The eight qPCR-typed individuals with uninterpretable kpi haplotype results had correct kpi gene calls, which could not be clearly assigned to a reference haplotype combination as provided by the software (Supplementary Table 4).

## Discussion

The broad availability of NGS data, generated in a multitude of clinical scenarios, allows for the inference of disease-relevant immunogenetic variation without additional dedicated typing efforts. Hence, in order to evaluate the usefulness in a clinical setting, we were interested in a systematic comparison of the newest generation of computational HLA typing solutions, run alongside the more well-established HLA typing tool Polysolver, that is limited in only typing HLA class I genes. Our analyses suggest that for both WES and WGS data, most of the tools outperform Polysolver in HLA inference, in both class I genotyping accuracy and the ability to perform inference on HLA II genes. For KIR typing using kpi, we are not aware of a published independent validation of its performance. Our evaluation demonstrates that kpi performs well at determining the absence or presence of a KIR gene, but it is not able to ascertain KIR allele or gene copy number.

Our analyses also show the breadth of class II genes that current state-of-the-art tools can infer. In particular, only HLA-HD was able to genotype the classical HLA II genes, *HLA-DRB3*, -*DRB4* and -*DRB5*, and we were able to further examine the results with our gold standard dataset. Interestingly, we found that many of the *HLA-DRB3*, *-DRB4* and *-DRB5* miscalls can be salvaged using knowledge of the strong (and clear) LD between the *HLA-DRB1* gene and its *DRB* paralogs [54]. It appears that incorporating this piece of biological information could be useful in developing tools that would like to genotype all the *DRB* genes, especially when there is high accuracy in genotyping the *HLA-DRB1*gene.

Additionally, with WGS and WES data for the same subjects, we observed that HLA inference from WGS data has yielded marginally higher accuracy compared to WES in many HLA genes (Tables 2 and 3). This possibly indicates that the addition of noncoding sequence information, or a more uniform read coverage in the HLA region, might be more relevant in these genes, especially in resolving alleles that are in the same G group, e.g. HLA-DQA1*03:01, -DQA1*03:02 and -DQA1*03:03. It might also point to the use of bait sets in WES, which can bias the calling of some alleles; WGS does not require such baits.

Of note, the dedicated HLA typing approach (LabCorp) identified one novel HLA-C allele in our cohort of 56 individuals at two-digit resolution. Even though all the tools gave an estimation (i.e. it was not a missing call) and were correct at the two-digit resolution, none of the tested tools were able to identify the allele as novel. This is because the inferences are all based on aligning sequencing reads to a database of known HLA alleles. This might not be highly important for large-scale genetic association approaches, but might be relevant in a clinical setting focused on individual patients, especially for ethnicities that have thus far been underrepresented with regard to genome sequencing, let alone HLA typing [55, 56].

The choice and accuracy of a given HLA method might depend on read length and sequencing coverage, which are factors that are not included in the current study. A recent comparison of HLA-HD and xHLA for use with target capture methods or amplification sequencing suggested that HLA-HD might decrease in sensitivity at read lengths below 150 base pairs (paired-end) [57].

We noticed that documentation for most of the HLA typing tools tested is mainly centered on the final inference, but not the auxiliary output files. The latter set of files typically contains the scores for all the candidates used for inference. Although accuracy is important, tool documentation in a clinical setting is also imperative to better understand the tool and its outputs, so that best practices can be developed in the clinic for different contexts.

As for the KIR typing efforts, kpi was shown to predict KIR gene presence/absence at >99% accuracy overall, and at >95% for each gene. Six out of nine errors were found in a single individual. This was likely due to a sample swap, and the remaining three miscalls were all for *KIR2DS3* in different patients. In this case, all other genes were inferred at 100% accuracy. However, since kpi detects gene presence/absence and does not perform an estimation of copy number, it assigns one or more possible haplotype combinations in many cases, resulting in considerable ambiguity. Thus, we recommend to analyze kpi results at the level of individual KIR genes, if possible. It is likely that the 25 uninterpretable haplotype pairs are due to carriers of rare haplotypes not present in the reference, which would prevent an assignment of possible reference haplotype combinations.

Notably, kpi requires WGS data and does not consider allelic variation within KIR genes. This is a significant limitation, since allotypes for a given KIR gene can be functionally different [58], and also have differential binding capacities to their predicted HLA ligands [49, 59, 60]. Allele-level typing would be desirable. The only software we are aware of that provides this level of granularity (PING) was not designed to work with NGS data in a high-throughput setting [61].

In conclusion, our survey for both high-resolution four-digit clinically relevant HLA typing and inference of KIR gene presence from NGS data (of conventional read length and coverage) indicated that recently published software tools can yield very high accuracy (>97% for HLA alleles and >95% for KIR genes, respectively), that may be suitable not only for research use, but also for the clinic. For comparison, the 2019 Standards for Accredited Laboratories issued by the ASHI requires that at least 80% of the samples are in full concordance with another CLIA-certified ASHI-accredited laboratory to be deemed satisfactory in clinical testing [62]. Most of the tools were able to achieve this, especially when using WGS data (Tables 2 and 3). It is noteworthy that WGS and WES continue to become less expensive, thereby presenting an alternative even in scenarios that focus only on HLA or KIR typing. However, a foreseeable hurdle is the process of obtaining regulatory approval for computational tools for HLA and KIR typing in the clinic, either as a stand-alone device, or as part of a pipeline. Such a process could be tricky as it can be highly dependent on the context of how the tool is being applied in the clinic. There are pros and cons for each tool. Other considerations in the choice of method that we did not explore in this study and might merit investigation in the future, include the characteristics of the NGS datasets at hand, such as the read length and read coverage, which can affect accuracy and thus cause deviations from what is shown in the present report. Also, although Illumina short-read sequencing is a standard in clinical sequencing, novel long-read technologies (as offered by e.g. Pacific Biosciences or Oxford Nanopore) have great potential in resolving complex genetic loci. A higher error rate [63] is accompanied with challenges for HLA or KIR typing efforts and will require dedicated computational approaches likely resulting in a new generation of software tools. But there are also clear advantages in long-read approaches, for example the ability to phase HLA haplotypes, as recently demonstrated in the context of a targeted sequencing approach [64].

Finally, we would like to further emphasize that computational tools can generate HLA and KIR information in a high-throughput manner on large cohorts of patients with clinical sequencing. Furthermore, the time and logistical challenges and risks associated with acquisition, preparation and shipping of valuable clinical specimens to perform a separate genotyping would be greatly reduced. In a clinical setting, the HLA and KIR results from these tools can then be applied directly to detect immunogenetic biomarkers that might be relevant for treatment decisions, or to predict the likelihood of adverse events for a given treatment of choice [65]. HLA typing is also a requirement in the context of individualized cancer treatment strategies, including immunization efforts and neoadjuvant-directed T-cell therapies [66, 67]. Neoepitope prediction requires highly accurate HLA types in order to maximize the likelihood of an immunogenic antitumor response [68]. KIR genes are emerging biomarkers in several disease areas, including cancer immunology [69], and should ideally be investigated in the context of their interactions with their HLA ligands. Having both HLA and KIR information will also allow stratification of patients according to their individual, and biologically relevant HLA–KIR interactions (demonstrated in Supplementary Figure 5) [22]. For example, it was shown that the presence of both *KIR2DL3* and *HLA-C1* ligands is associated with increased Hepatitis C virus clearance [70, 71]. The ability to investigate immunogenetic variation without having to engage in dedicated typing efforts might stimulate hypothesis generation and facilitate similar discoveries in the future. Finally, as more computational tools for HLA and KIR typing are likely being developed in the future, they should be continuously evaluated so that they can fulfill a greater role in assessing clinical genomes.

**Key Points**

- *In silico* typing of HLA and KIR holds the potential to enable large-scale analyses in research settings, as well as facilitate and accelerate clinical decision making.
- Recently published software tools for HLA typing from WGS and WES data are highly accurate for both class I and class II genes.
- A recently published tool for KIR haplotype inference accurately predicts KIR gene presence/absence, but does not consider allelic variation.
- Availability of both HLA and KIR types allows the coding of experimentally verified interactions and testing of biological hypotheses related to NK cell activity.

## Supplementary Material

supplement_revised_bbaa223

## Notes

**Jieming Chen**, PhD, is an associate scientist in the Department of Bioinformatics and Computational Biology at Genentech.

**Shravan Madireddi**, PhD, is a senior scientific researcher in the Department of Cancer Immunology at Genentech.

**Deepti Nagarkar**, PhD, is a scientific manager in the Department of Cancer Immunology at Genentech.

**Maciej Migdal** is a PhD candidate, and a bioinformatics contractor at Roche’s Global IT Solution Centre.

**Jason Vander Heiden**, PhD, is a scientist in the Department of Bioinformatics and Computational Biology at Genentech.

**Diana Chang**, PhD, is a scientist in the Department of Human Genetics at Genentech.

**Kiran Mukhyala**, MSc, is a Sr. computational biologist and bioinformatics engineer in the Department of Bioinformatics and Computational Biology at Genentech.

**Suresh Selvaraj**, PhD, is a scientific manager in Roche/Genentech’s Biosample & Repository Management.

**Edward E Kadel III**, BS, is a principal scientific researcher in the Department of Oncology Biomarker Development at Genentech.

**Matthew J. Brauer**, PhD, is vice president for Data Science at Maze Therapeutics.

**Sanjeev Mariathasan**, PhD, is a principal scientist and associate director in the Department of Oncology Biomarker Development at Genentech.

**Julie Hunkapiller**, PhD, is a senior scientific manager in the Department of Human Genetics at Genentech.

**Suchit Jhunjhunwala** PhD is a scientist in the Department of Bioinformatics and Computational Biology at Genentech.

**Matthew L. Albert**, MD, PhD, is vice president of Immunology & Infectious Diseases at Insitro.

**Christian Hammer**, PhD, is a scientist in the Departments of Cancer Immunology and Human Genetics at Genentech.
